# Terrorism’s Impact on Mental Health Outcomes among Directly and Indirectly Exposed Victims and the Development of Psychopathology

**DOI:** 10.3390/jcm11092630

**Published:** 2022-05-07

**Authors:** Dariusz Wojciech Mazurkiewicz, Jolanta Strzelecka, Dorota Izabela Piechocka

**Affiliations:** 1 St. Mark’s Place Institute for Mental Health, 57 St. Mark’s Place, New York, NY 10003, USA; 2 Department of Pediatric Neurology, Medical University of Warsaw, Al. Żwirki and Wigury 63A Street, 02-091 Warsaw, Poland; jolanta.strzelecka@wum.edu.pl; 3 Department of Gynecology and Practical Obstetrics, Medical University of Bialystok, Szpitalna 37 Street, 15-295 Bialystok, Poland; dorota.piechocka@umb.edu.pl

**Keywords:** terrorism, psychopathology, trauma, PTSD, victims

## Abstract

After the events of 9/11, many police-responders developed post-traumatic stress disorder (PTSD) and were potentially vulnerable to developing depression and/or anxiety; in addition, nearly half of police with probable PTSD had comorbid depression and anxiety. Having in mind that victims who experience the effects of terrorism are exposed to high levels of psychological damage, we thus aimed to determine how sequelae of a terrorist act directly and indirectly affect victims. Quantitative synthesis findings were concluded on the basis of 200 records that met the inclusion criteria out of a total of 650. We grouped the patients according to their level of exposure to the WTC terrorist attack on 11 September 2001. The Level I group included individuals who had experienced the traumatic event and/or those who had observed the attack. The Level II group consisted of rescuers and/or persons who cleaned up debris in the area after the attack. The Level III group comprised the victims’ families. Our research enabled us to create a profile for those who were most vulnerable to mental disorders after the WTC terrorist attack. Patients who had survived the terrorist attack and/or those who had observed the incident exhibited fewer traumatic symptoms and a lower percentage of suicidal thoughts in comparison to individuals who had worked as rescuers or cleaning staff in the area after the attack. The number of symptoms rose along with increased contact time with the stressor. The dominant symptom was the triad of intrusion, avoidance, and hyperarousal. The findings may confirm the positive effect of protracted court cases in legal proceedings for compensation on the maintenance and development of psychopathology. Our research may contribute to a better understanding of the consequences of terrorism outcomes on the human psyche and be used in the development of standards for dealing with victims of terrorism’s impact.

## 1. Introduction

In the years after the terrorist attack on the World Trade Center (WTC) in New York, the health consequences for the direct and indirect victims of this event remain visible; the primary symptoms of mental disorders are post-traumatic stress disorder (PTSD), depression, and somatic complaints (such as shallow respiratory tract, gastrointestinal reflux, and asthma) [1]. In the Stony Brook cohort, 17% of responders developed PTSD in the first 13 years after the attack, and half developed an active illness 11–13 years later [2]. PTSD is a risk factor for respiratory symptoms and is consistent with evidence implicating physiological dysregulation associated with PTSD in the development of medical conditions [3]. PTSD may be a causal risk factor for subsequent depression [4]. The number of cases registered before 30 June 2016 in the WTC Health Program—including mental and somatic diseases among unskilled workers, volunteers, paramedics, police officers, and firemen—is high in its quantitative distribution. The most frequently identified diagnoses include the following: PTSD (7160 patients), depression (2810 patients), and generalized anxiety disorder (GAD, 2451 patients). According to the Top 10 Certified Conditions of the World Trade Center Health Program 2016, including 9/11 respondents and survivors, mental illness (12,500 patients) ranks second after 32,289 patients with respiratory and gastrointestinal diseases, where in fact 9/11 respondents and survivors had never suffered from such conditions before 11 September 2001 [5]. On 30 September 2017, the number of deceased first responders (in comparison to living first responders) was reported due to poor health conditions in the following categories: aerodigestive issues (345 of 29,380); mental health (136 of 10,222); and cancer (253 of 6221) [6]. 

The mental health symptoms of WTC tower survivors (compared to other survivors) a decade after the attack include binge drinking, as well as the influence of infrastructure and behavioral barriers during the evacuation, revealing a significant association with PTSD. Infrastructure barriers are those inherent in the structural environment and architecture of the building, and include number and spacing of floors, configuration of stairways and exits, and damage-related conditions hindering or preventing exit such as fire and water conditions. Behavioral barriers include crowding, panic, perception of danger, and communication problems. PTSD and frequent binge drinking were associated with being in the towers but not being in other buildings, compared to persons on the street. Among those who evacuated a building (WTC 1 and 2 or other buildings) on 9/11, those who reported infrastructure and behavioral barriers were significantly more likely to have PTSD. Those who faced barriers to evacuation may have experienced additional fear for their lives or safety during evacuation, potentially leading to higher risk of PTSD. [7] 

Exposure to the 9/11 disaster has had long-term effects for survivors and responders regarding depression up to 15 years later [8], and a significant number of 9/11-exposed persons continue to experience PTSD symptoms and engage in intentional self-medication with alcohol among this group as a potential coping mechanism [9]. Comparable findings between responders and civilians suggest that 9/11-related PTSD is associated with an increased mortality risk [10,11]. A few studies have examined the incidence of confusion or memory loss or its association with mental health in 9/11 attack survivors [12]. Interventions focusing on anxiety sensitivity reduction (specifically addressing physical concerns about bodily sensations) may be useful in addressing elevated BMI among trauma-exposed persons [13]. After the 9/11 event, many police-responders developed PTSD and might be vulnerable to developing depression and/or anxiety; in addition, nearly half of police with probable PTSD had comorbid depression and anxiety [14]. Findings suggest that WTC exposure may compound post-disaster life stress, thereby resulting in a more chronic course of PTSD symptoms and reduced functioning among police responders [15]. In general, the influence of severe exposures, scarce personal/financial resources, and treatment barriers on PTSD trajectories suggest a need for early and ongoing PTSD screening post disaster [16]. However, prolonged work at the WTC site, independent of acute exposure, was an important predictor of post-9/11 systemic autoimmune diseases [17]. An injury, losing someone, and witnessing horror were the three most pernicious exposures and these three exposures should be a particular focus in psychological evaluation and treatment programs’ intervention and future emergency preparedness efforts [18]. Having in mind that victims who experience the ramifications of terrorism are exposed to high levels of psychological damage, we thus aimed to determine how sequelae of a terrorist act directly and indirectly affect victims. Our research may contribute to a better understanding of the consequences of terrorism outcomes on the human psyche and be used in the development of standards for dealing with victims of terrorism’s impact.

## 2. Materials and Method

The research was conducted based on an analysis of the source materials concerning mental health records of the WTC terrorist attack victims, which are in the possession of St. Mark’s Place Institute for Mental Health, doing business as (d/b/a) Unitas.

### 2.1. Inclusion and Exclusion Criteria

The inclusion criteria related to WTC terrorist attack sequelae were as follows: (1) clinical diagnosis and symptoms; (2) a triad of intrusion, avoidance, and hyperarousal; (3) levels of exposure to the attack: individuals who had experienced the traumatic event and/or those who had observed the attack; (4) contact time with the stressor; (5) the initiation of conflict situations; (6) an individual’s social, occupational, and psychological functioning in everyday life; (7) range of age 5–65; (8) medical and psychological records treated between 2001 and 2008; (9) rescuers and/or persons who cleaned up debris in the area after the attack; (10) members of the victims’ families.

### 2.2. Participants 

Quantitative synthesis findings were concluded on the basis of 200 records that met the inclusion criteria out of a total of 650. This allowed us to select 200 patients (171 males and 29 females) who reported their symptoms individually or who were referred from other medical centers between 2002 and 2008 due to mental disorders following the WTC terrorist attack on 11 September 2001. The male and female groups ranged in age from 21–32 years, 33–34 years, 44–54 years, to 55–56 years. The group that ranged in age from 5 to 11 years was identified in the male group only.

### 2.3. Procedure and Data Collection

We grouped the patients according to their level of exposure to the WTC terrorist attack on 11 September 2001. The Level I group (*n* = 31) included individuals who had experienced the traumatic event and/or those who had observed the attack. The Level II group (*n* = 180) consisted of rescuers and/or persons who cleaned up debris in the area after the attack. The Level III group (*n* = 4) comprised the victims’ families. The data were collected in worksheets as follows:

#### 2.3.1. Worksheet to Analyze the Occurrence of Mental Disorders in WTC Victims and According to the Exposure Levels

Included data were as follows: documentation code, exposure level (Level I, Level II, Level III), sex (male, female), age of the patient, time of contact with the stressor, time from the onset of contact with the stressor to the first symptoms, diagnosis according to DSM-IV classification (Axis I, Axis II, Axis III, Axis IV), date of diagnosis.

#### 2.3.2. Worksheet for a Comparative Analysis of Symptoms of Mental Disorders in WTC Victims and According to the Exposure Levels

Included data were as follows: documentation code, exposure level (Level I, Level II, Level III), age of the patient, sex (male, female), symptoms.

#### 2.3.3. Worksheet for the Analysis of the Length of the Period of Psychological Therapy and Psychiatric Treatment 

Included data were as follows: documentation code, age of the patient at the onset of contact with the stressor, length of time from the initiation of contact with the stressor to date of diagnosis, date of diagnosis, diagnosis according to DSM-IV, psychological therapy (sex: male, female; date of beginning/endings; total time of treatment), psychiatric treatment (sex: male, female; date of beginning/endings, total time of treatment).

#### 2.3.4. Worksheet for the Analysis of the Effectiveness of Pharmacological Therapy in the Treatment of Mental Disorders of WTC Victims

Included data were as follows: documentation code, age of the patient, sex (male, female), drug name, drug dosage (date of beginning/ending use, initial dose, dose change, date of dose change or discontinuation), achieved therapeutic effectiveness of the drug (week, quarter).

#### 2.3.5. Worksheet for the Analysis of the Correlation between Psychological Therapy and Psychological Treatment and Obtaining Compensation in Judicial Compensation Proceedings

Included data were as follows: documentation code, date of commencement (psychological therapy, psychiatric treatment), date of completion (psychological therapy, psychiatric treatment), status of therapy/treatment achievement (achieved, not achieved), compensation for workplace health impairment (called “Workers” Compensation) and/or American Red Cross financial assistance: date of application, date of award of compensation or reason for denial, duration of compensation processes.

We subjected the data, compiled in our own data collection sheets, to statistical analysis.

### 2.4. Data Analysis

We performed statistical analysis with IBM SPSS 24.0, configured as PS IMAGO 4.0 (license No. 2971), with the R environment extension using Microsoft Office 2013. We applied the following statistical methods: frequency tables, contingency tables, chi-square tests, Spearman’s rho correlation coefficients, scatter plot graphs, Mann–Whitney U tests, Kruskal–Wallis tests, and box plot graphs. In the case of quantitative variables, we applied descriptive statistics, specifically the median, arithmetic mean, minimum, maximum, standard deviation, and number.

## 3. Results

Our research study analyzes the presence of terrorism’s impact on mental health outcomes among directly and indirectly exposed victims and the development of psychopathology. The findings allows for us to divide and describe findings in ten domains as follows.

### 3.1. Domain 1: Clinical Diagnosis and Symptoms

We examined 52 clinical diagnoses and 22 symptoms. We detected the convergence and divergence of the type of diagnosis determined for the gender criterion; some diagnoses made by clinicians are attributed only to males, not females, and vice versa. The convergence and divergence refer to Axis I, Axis II, Axis III, and Axis IV diagnoses. The diagnoses were selected based on the DSM-IV classification (in force from 2000 to 2012), determined only for females, for males, and diagnoses established independently of patients’ gender. The most common Axis I diagnoses are PTSD; severe depressive disorder, first episode; dysthymia; and generalized anxiety disorder (GAD). The most common Axis II diagnoses are borderline personality disorder, and personality disorder not otherwise specified. The most common Axis III diagnoses reserved for medical or physical conditions are cardiovascular disorders, asthma, gastritis, hypercholesterolemia, and arterial hypertension. The most common Axis IV diagnoses are social isolation/no friends, family conflict, no financial stability/unemployment, no support from family/friends, traumatic recollections of Ground Zero, and a health-related incapacity to work.

The results provide knowledge about an extremely strong and traumatic stressor impact (the stressor being the WTC terrorist attack) on human body dysfunction, primarily in the mental domain, which in most cases is reflected in a clinical expression, including in recurring memories of traumatic events (flashbacks), avoidance symptoms (evading conversations and thoughts about 9/11, as well as the WTC site or people who bring back memories of the attack), emotional numbness (including anhedonia, a sense of alienation, reduced expression of emotions, indifference toward the future, and retrograde amnesia), and anxiety disorders.

### 3.2. Domain 2: A Triad of Intrusion, Avoidance, and Hyperarousal

Dominant among the symptoms was the characteristic triad of intrusion, avoidance, and hyperarousal. Pearson’s chi-square test showed statistically significant differences between males and females regarding irritation (*p* = 0.027), which was more common in males, and in terms of concentration disorders (*p* = 0.024), which were more common in females. We found statistically significant differences between males and females (*p* = 0.039) in the analysis of a state of irritation and anger. All analyzed states of irritation, anger, and the combined occurrence of irritation and anger more frequently concerned men than women.

### 3.3. Domain 3: The Occurrence of Differences in the Types of Symptoms and the Frequency of the Respective Symptoms between Levels of Exposure to the Attack

Given the occurrence of differences in the types of symptoms and the frequency of the respective psychological symptoms between Levels I and II of exposure to the WTC terrorist attack and the aftermath of mental health disorders, we found statistically significant differences between the groups among men (*p* < 0.0005) and in the entire group (*p* < 0.0005) since anger and panic attacks were more common in people at Level I, and depressed mood was more common in people at Level II.

### 3.4. Domain 4: The Effect of Length of Contact with the Stressor on the Incidence of Symptoms

**Figure 1:**
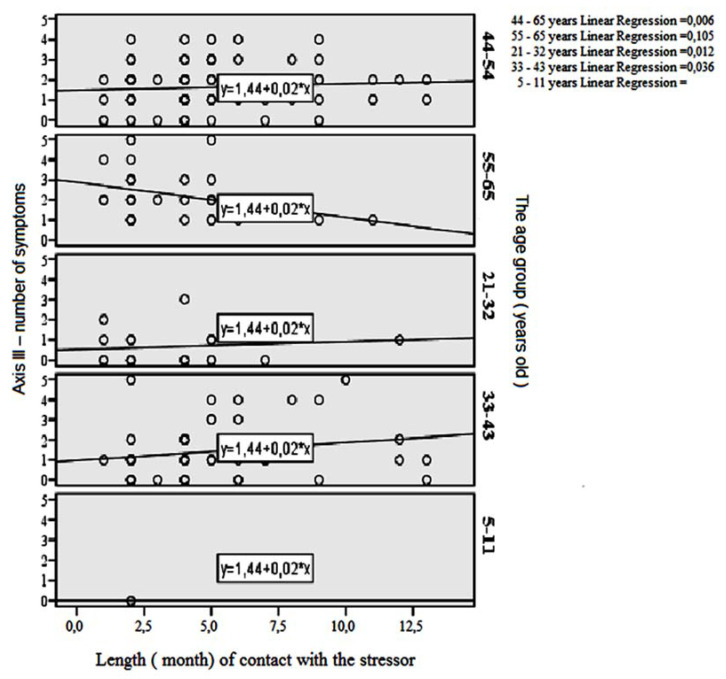
Length of contact with the stressor in the scatter plot.

With regard to the effect of length of contact time with the stressor (taking into account patients’ age) on the incidence of the respective symptoms of Axis I, Axis II, Axis III, and Axis IV, the application of symmetric measures for Axis I, Axis II, Axis III and Axis IV demonstrated a statistically significant, positive relationship among 33–43-year-olds, as well as in the entire population. The number of symptoms rose along with increased contact time with the stressor. A positive relationship between the variables ‘Axis III—number of symptoms’ and ‘Length of contact with the stressor’ in the scatter plot (Figure 1) emerged in the age groups of 33–43, 21–32, and 44–54. A negative relationship occurred in the 55–65 age group.

We observed a statistically significant, negative relationship between contact time with the stressor and the number of Axis IV symptoms in the age groups of 21–32 and 55–65, where the number of symptoms fell along with increased contact time with the stressor (Table 1).

### 3.5. Domain 5: The Experience of a Massive Terrorist Attack and the Initiation of Conflict Situations

For Axis IV, as for the problem/symptom ‘No financial stability/unemployment’, we found statistically significant relationships between the variables in the age group ≤46 years. Among people with a shorter contact time with the stressor, symptoms appeared more rarely. The experience of a massive terrorist attack is responsible for the initiation of conflict situations, resulting in Axis IV psychosocial stressors: an increased number of homeless people (males aged 33–43) and family conflicts (males of all ages, excluding those aged 5–11; females aged 33–43 and 44–54); conflicts with the law (only males aged 44–54 and 55–65); marital conflicts including marriage breakdowns (only males aged 33–43 and 44–54); and job loss and increased unemployment in all female and male age groups (excluding the 5–11 age group). All the above identified psychosocial stressors are responsible for the initiation of personal and/or family conflict situations.

### 3.6. Domain 6: The Distribution between the Time of Initiation of Contact with the Stressor and the Onset of the First Symptoms of Victims

**Figure 2:**
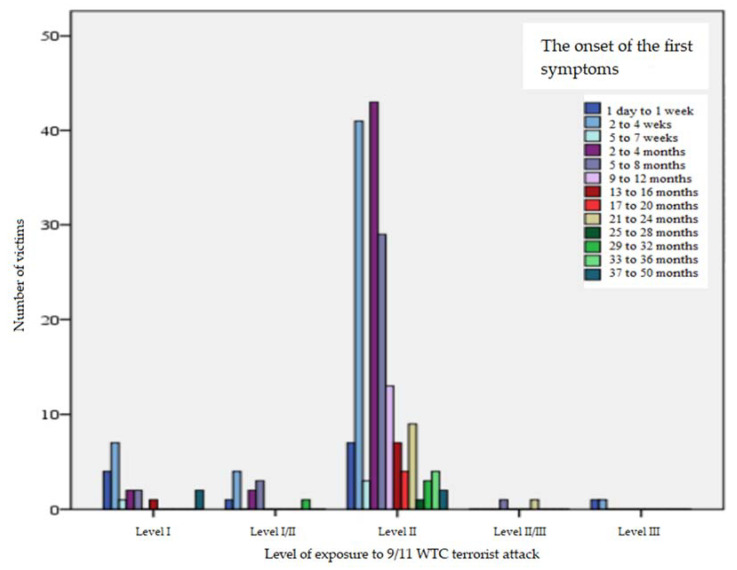
The distribution between the time of initiation of contact with the stressor and the onset of the first symptoms of victims.

We established the distribution between the time of initiation of contact with the stressor and the onset of the first symptoms of victims (Figure 2). This range varies from 1 day to 50 months. The greatest frequency of the first symptoms emerged within 2–4 months after experiencing the attack; a slightly lower frequency appeared in within 2–4 weeks; and an even lower frequency showed up within 5–8 months. The largest time diversity (in months)—taking into account the two variables considered (the relationship between time from initiation of contact with the stressor and the onset of the first symptoms of victims)—occurred among victims with Level II exposure in relation to mental disorders. In most cases, the first symptoms emerged at the latest in the families of the victims (Level III exposure to the attack), as well as in attack survivors and/or people who had observed the act of terror (Level I exposure to the attack).

We witnessed statistically significant relationships (between gender and the level of exposure to the attack) between the variables (*p* < 0.0005). Among females, the largest number exhibited Level I; among males, it was Level II.

The patients’ age affected the level of exposure in relation to mental disorders (excluding coincidences) due to the following statistically significant relationships between the variables (*p* < 0.0005). Among those with Level II exposure, those over 33 years of age prevailed.

### 3.7. Domain 7: The Profile of Those Most Vulnerable to Mental Disorders after the WTC Terrorist Attack

Our research enabled us to create a profile for the individuals who were most vulnerable to mental disorders after the WTC terrorist attack. Such people are male in the age group of 33–43 (and/or 44–54), and belong to the group with Level II exposure to the attack (i.e., a rescuer and/or person who cleaned up debris in the area after the attack).

The patients who had survived the attack and/or people who had observed the incident exhibited fewer traumatic symptoms and a lower percentage of suicidal thoughts in comparison to the group of individuals who had worked as rescuers or staff cleaning the area after the attack.

### 3.8. Domain 8: The Duration of Individual or Group Psychotherapy

We did not detect any statistically significant differences between females and males regarding the duration of individual or group psychotherapy. Statistically significant differences among the age groups occurred regarding the duration of individual psychotherapy (*p* = 0.002). The longest duration of therapy was in the 55–65 age group (from 0.30 to 7.70 years) and in the 33–43 age group (from 0.14 to 0.31 years).

There were statistically significant differences among age groups (*p* = 0.002). We noted the longest duration of psychiatric treatment in the 33–43 age group (from 1.01 to 4.60 years); the shortest duration was in the 21–32 age group (0.16–0.77 years).

We established statistically significant differences between females and males regarding frequency distribution (*p* = 0.025). Females initiated psychological treatment more often in 2002, 2003, 2006, and 2007. The percentage of males was predominant in 2004 and 2005.

The largest share of individuals started psychiatric treatment at St. Mark’s Place Institute for Mental Health from 2004 to 2006 (3–5 years after the attack). This is justified by the scientific literature, which confirmed that in victims with no history of PTSD, symptoms may appear 5–6 years after a terrorist attack.

### 3.9. Domain 9: An Individual’s Social, Occupational, and Psychological Functioning in Everyday Life

As for Axis V, a numeric scale that allows one to subjectively rate his/her social, occupational, and psychological functioning in everyday life (Global Assessment of Functioning (GAF) score), we noted the following values on the scale: 30 was the lowest (0.5% of subjects), rising to 40 (5% of subjects), 50 (40% of subjects), 55 (10% of subjects), 60 (29.5% of subjects), and 70 (15% of subjects).

### 3.10. Domain 10: The Effect of Protracted Court Cases in Legal Proceedings for Compensation on the Maintenance and Development of Psychopathology

A total of 18.6% of people who sought compensation gave up treatment a few days after being denied compensation, which may confirm the positive effect of protracted court cases in legal proceedings for compensation on the maintenance and development of psychopathology. We observed statistically significant differences among the groups (Z = −1.964; *p* = 0.049). People who gave up treatment a few days after being denied compensation experienced a shorter duration of the compensation process. The analysis concerned respondents with an attitude of entitlement who failed to carry out the treatment plan since they gave up on treatment in response to having been denied financial aid.

## 4. Discussion

Our findings undoubtedly prove that the group exposed to the psychiatric and psychological effects of the WTC attack includes not only victims who were directly exposed to the attack, but also the individuals who worked as medical staff/rescuers or cleaning staff afterward. The results provide knowledge about an extremely strong and traumatic stressor impact (the stressor being the WTC terrorist attack) on human body dysfunction, primarily in the mental domain, which in most cases is reflected in a clinical expression. The study’s outcomes are positively correlated with the scientific publications of other authors who have revealed that 10 and 15 years after the WTC terrorist attack, medical staff who provided medical services to victims started to experience nightmares, intrusive memories (from the WTC site where help was rendered), PTSD, anxiety, depression, sleep disorders, as well as negative effects on family and marital relationships and civil unions, where the central somatic problems are respiratory disorders, GARD, eyesight and eye problems, and neoplasms [1,19,20].

The state of nervous tension and hyperactivity may be symptoms typical of re-experiencing traumatic events, including the WTC terrorist attack, whereby symptoms of dysphoric arousal, associated with a sense of dissatisfaction with life (which is common in concentration disorders), a state of irritability, sleep disorders, and anger may be responsible for a state of emotional numbness [21]. We confirmed these symptoms in the statistical part of our research, especially in terms of classification:

- Hyperactivity in women for Level I exposure (over 20%); for Level II exposure, nearly 38%; for Level III exposure, hyperactivity was not exhibited.
- In the classification of symptoms for the gender criterion, this figure was almost 25% for hyperactivity in men. For Levels I and II of exposure, it was 20%. In the classification of the discussed symptoms for the gender criterion, it was slightly over 19%.
- Women had difficulty concentrating. For Level 1 exposure, this was approximately 33%; for Level II exposure: 50%. For Level III exposure, no women had problems focusing. In the classification of symptoms for the gender criterion, this figure was almost 45%.
- Men had problems concentrating. For Level I exposure, this was 20%; for Level II exposure: just over 25%. For Level III exposure: men had no problems concentrating. In the classification of symptoms for the gender criterion, this figure was approximately 25%.
- As for the state of irritation in women, for Level I exposure, it was about 44%; for Level II exposure: almost 38%. For Level III exposure, women’s irritation status was not confirmed. In the classification of symptoms for the gender criterion, this figure was approximately 59%.
- Regarding the state of irritation in men, for Level I exposure, it was 70%; for Level II exposure: 63.3%. For Level III exposure, men’s irritation status was not confirmed. In the classification of symptoms for the gender criterion, this figure was 63.2%.
- Women experienced insomnia and sleep problems. For Level I exposure, this was almost 56%; for Level II exposure: 87.5%. For Level III exposure, insomnia and sleep problems among women were not confirmed. In the classification of the discussed symptoms for the gender criterion, this figure was 75.9%.
- Men also experienced insomnia and sleep problems. For Level I exposure, this figure was exactly 70%; for Level II exposure: 76.7%. For Level III exposure, it was 50%. In the classification of symptoms for the gender criterion, the figure was 75.4%.
- Women experienced anger. For Levels I, II, and III of exposure, women did not exhibit any symptoms of anger. In the classification of symptoms for the gender criterion, this figure was 3.4%.

Male anger was also a factor. For Level I exposure, it was 60%; for Level II exposure: 14.7%. For Level III exposure, men had no symptoms of anger. In the classification of symptoms for the gender criterion, this figure was 16.4%.

The outcomes of Pearson’s chi-square test did not show statistically significant differences between women and men in terms of anger symptoms or insomnia and sleep disorders. Pearson’s chi-square test indicated statistically significant differences between women and men in terms of irritation; irritation was more common in men. Pearson’s chi-square test revealed statistically significant differences between men and women regarding concentration disorders; concentration disorders were more common in women. Pearson’s chi-square test did not point to statistically significant differences between women and men in the frequency of hyperactivity.

The lack of evidence of statistically significant differences between women and men related to symptoms of anger is somewhat discordant with Evans et al. [22], assuming that a higher level of anger should be observed in respondents who have been diagnosed with PTSD. However, Evans et al. [22] did not solve the issue of what level of anger should be expected in comparably high percentages of PTSD occurrence in women and men. Bearing in mind the data from the analysis of the frequency of individual symptoms of Axis I in age criterion groups, with the preservation of the gender criterion for PTSD (the subject of research in our study), the prevalence of PTSD in women at the level of 41% should be assumed for the 44–54 age group, and in men at the frequency level of 44,1%. Since the PTSD frequencies in both groups were high and comparable, concluding with the assumptions of Evans et al. [22], we can assume this fact is the reason for the lack of statistically significant differences between women and men related to symptoms of anger. There were statistically significant differences between women and men (*p* = 0.039) in the analysis of the complex symptoms of mental disorders, specifically a state of irritation and anger. The state of irritation, anger, and the total occurrence of irritation and anger are more likely to affect men than women.

The abovementioned symptoms fall under adaptive disorders. In the case of a traumatic event such as the WTC terrorist attack, we must distinguish these symptoms from (among others) PTSD, depressive syndrome, acute stress reaction, and dysthymia.

The following are diagnostic elements in PTSD: intrusion (nightmares; flashbacks; auditory, visual, and olfactory hallucinations/sensations), avoidance (in withdrawal, for example, isolation from people and activities that used to bring pleasure), or excessive excitation/increased alertness. In the clinical picture, excessive excitation/increased alertness often manifests as the symptom of a sleep disorder, state of irritation, or concentration disorder, in connection with trauma resulting in anxiety and/or horror, and persisting for a period of at least one month. This is considered a criterion for identifying PTSD; at least one intruder symptom must be confirmed, as well as two symptoms of increased excitation, and three symptoms of avoidance [23,24]. Referring to the PTSD diagnostic criteria laid out above, we carried out medical-psychological documentation regarding a group of 200 patients who received care at St. Mark’s Place Institute for Mental Health; this provided knowledge, among other things, about recurring facial expressions tied to unpleasant traumatic sensations, or recurring flashbacks. Avoidance symptoms (evading conversations and thoughts about 9/11 and sites or people reminiscent of the attack), emotional numbness (taking into account a lack of pleasure, a sense of alienation, a constraint on expressing one’s emotions, feelings of indifference about the future, and retrograde remission), anxiety disorders, and other PTSD symptoms positively correlated with, inter alia, the work of Malta et al. [25].

Turning toward depression in the discussion, it is necessary to underscore the much higher frequency of clinical depression in patients who survived the WTC attack (Level I exposure), which is derived from the investigation of documentation and statistical analyses published by Salguero et al. [26]. Salguero et al. suggested the incidence of clinical depression in 20–30% of people who have been direct victims of a terrorist attack. For example, we confirmed the incidence of depressive disorders with psychotic episodes in the male age groups of 33–43 and 55–65 at the 50% level, and a 50% incidence of unspecified depressive disorders among men aged 44–54.

The examination of the medical-psychological documentation of the research group makes it possible to conclude that if the first contact with the stressor is delayed by a minimum of four months from its peak (the day of the WTC attack), this may shorten the period of psychotherapy in individual cases, and often results in a lack of medication prescribed in psychiatric treatment. On the other hand, Yip et al. [27] showed that early arrival at work and/or prolonged work at the WTC site increased the risks for adverse physical and mental health outcomes.

In addition, the present study allowed us to create a profile for those who were most vulnerable to mental disorders after the attack. Such people are male in the age group of 33–54 with Level II exposure (i.e., a rescuer, and/or person who cleaned debris in the area after the attack). This profile corresponds to reports by Chen et al. [28], who found that 40-year-olds and 50-year-olds were the most exposed to emotional disturbances due to 9/11; the least exposed were older people and those under 40.

The highest percentage values of the number of patients (the research group) starting psychiatric treatment at St. Mark’s Place Institute for Mental Health was recorded in 2004–2006. This verifies scientific data that symptoms can appear 5–6 years after an attack in victims who do not have a history of PTSD [29].

The rise in the number of PTSD diagnoses in rescuers and people who cleaned up debris a few years after the WTC attack correlates with the rise in PTSD diagnoses among soldiers who have taken part in military operations in Afghanistan for five years from their participation [30]. Rescue workers generally have a much higher prevalence of PTSD compared to the general population, since their duties routinely entail facing traumatic stressors [31,32]. Disaster preparedness training and shift rotations to enable a shorter duration of service at a site may reduce PTSD among workers and volunteers in future disasters [33]. The role of emergency response training in preventing disaster-related mental illness should be explored as a possible strategy to improve resilience to disaster events [34]. In our opinion, further active research must consider the long-term effects of terrorism on human mental stability, as well as the need to develop standards to prevent and treat mental disorders following acts of mass terrorism on direct and indirect victims.

### Strengths and Limitations

We used statistical methods to obtain reliable results on a group of 200 patients (171 males and 29 females). The guidelines for the care of victims of terrorist attacks, their relatives, witnesses, and workers in areas where such events occur can be enriched with this type of research. Data published in Psychiatry Resources, the Journal of Traumatic Stress, and the Journal of Affective Disorders suggest a discordance between the previous version of the definition of PTSD in the DSM-IV and the newer DSM-V [35,36,37,38]. The DSM-V contains important modifications to the diagnostic guidelines for PTSD. For example, the diagnosis of comorbidity with depression invariably became minimized with the release of the DSM-V given the new formulation for PTSD, which includes symptoms of anhedonia and depressive mood. It is necessary to alert readers about such changes, and to consider such aspects for debates that involve preventive and curative work in PTSD; this requires collaboration between clinics, institutions of justice, the police, and social services. We prepared this manuscript using a research-based analysis of the source materials (2001–2008), which consisted of victims’ medical and psychological records regarding their levels of exposure to the WTC attack. We examined the data using the DSM-IV classification, which went into effect in 2000–2012. Hence, the goal is not to refer to the results and/or diagnosis and/or symptoms based on the DSM-V classification, which did not exist in 2001–2008. Otherwise, our study would lack an ethical scientific manner, since the symptoms and diagnoses in the records from St. Mark’s Place Institute for Mental Health are based on the DSM-IV (the classification was binding until 2012), and not on the DSM-V.

## 5. Conclusions

The number of symptoms rose along with increased contact time with the stressor. The dominant symptom was the triad of intrusion, avoidance, and hyperarousal. The gender criterion is related to the occurrence of differences in the types of symptoms of mental disorders resulting from a massive terrorist attack. Our research enabled us to create a profile for those who were most vulnerable to mental disorders after the WTC terrorist attack. Patients who had survived the terrorist attack and/or those who had observed the incident exhibited fewer traumatic symptoms and a lower percentage of suicidal thoughts in comparison to individuals who had worked as rescuers or cleaning staff in the area after the attack. The findings may confirm the positive effect of protracted court cases in legal proceedings for compensation on the maintenance and development of psychopathology.

## Figures and Tables

**Table 1:** The effect of contact with the stressor on the incidence of Axis I–IV symptoms in the age group.

	*S* *ymmetric Measures*		Value	*p*	Value	*p*	Value	*p*	Value	*p*
The Age Group			Axis I	Axis I	Axis II	Axis II	Axis III	Axis III	Axis IV	Axis IV
5–11 years		N	2		2		2		2	
21–32 years	Spearman’s rho correlation		−0.074	0.744	−0.316	0.151	0.259	0.245	−0.247	0.267
		N	22		22		22		22	
33–43 years	Spearman’s rho correlation		0.011	0.938	−0.026	0.849	0.309	0.023	0.157	0.257
		N	54		54		54		54	
44–54 years	Spearman’s rho correlation		0.011	0.915	0.078	0.461	0.267	0.011	0.183	0.082
		N	91		91		91		91	
55–65 years	Spearman’s rho correlation		−0.379	0.036	−0.128	0.492	−0.196	0.290	−0.097	0.603
		N	31		31		31		31	
Total	Spearman’s rho correlation		−0.042	0.551	0.020	0.774	0.239	0.001	0.095	0.182
		N	200		200		200		200	
